# Rule extraction from biased random forest and fuzzy support vector machine for early diagnosis of diabetes

**DOI:** 10.1038/s41598-022-14143-8

**Published:** 2022-06-14

**Authors:** Jingwei Hao, Senlin Luo, Limin Pan

**Affiliations:** Information System and Security and Countermeasures Experiments Center, Beijing Institute of Technology, Beijing, 100081 People’s Republic of China

**Keywords:** Data mining, Data processing, Machine learning, Probabilistic data networks

## Abstract

Due to concealed initial symptoms, many diabetic patients are not diagnosed in time, which delays treatment. Machine learning methods have been applied to increase the diagnosis rate, but most of them are black boxes lacking interpretability. Rule extraction is usually used to turn on the black box. As the number of diabetic patients is far less than that of healthy people, the rules obtained by the existing rule extraction methods tend to identify healthy people rather than diabetic patients. To address the problem, a method for extracting reduced rules based on biased random forest and fuzzy support vector machine is proposed. Biased random forest uses the k-nearest neighbor (k-NN) algorithm to identify critical samples and generates more trees that tend to diagnose diabetes based on critical samples to improve the tendency of the generated rules for diabetic patients. In addition, the conditions and rules are reduced based on the error rate and coverage rate to enhance interpretability. Experiments on the Diabetes Medical Examination Data collected by Beijing Hospital (DMED-BH) dataset demonstrate that the proposed approach has outstanding results (MCC = 0.8802) when the rules are similar in number. Moreover, experiments on the Pima Indian Diabetes (PID) and China Health and Nutrition Survey (CHNS) datasets prove the generalization of the proposed method.

## Introduction

The incidence of diabetes is increasing in the world and has become a major global public health problem in the twenty-first century. Diabetes is a common chronic disease characterized by chronic hyperglycemia with carbohydrate, fat and protein metabolic disorders, which is caused by insulin secretion deficiency or insulin action deficiency. Diabetes is usually divided into type I diabetes mellitus (T1DM), type II diabetes mellitus (T2DM) and gestational diabetes mellitus. Among them, the cause of T2DM patients is mainly insulin resistance, accompanied by insufficient insulin secretion, accounting for 90–95% of diabetic patients^1^. According to the latest statistics from the International Diabetes Federation (IDF), approximately 8.8% of adults aged 20–79 have diabetes worldwide, of which 46.5% are undiagnosed, and approximately 5 million die each year from diabetes^2^. By 2040, the number of people suffering from diabetes is expected to reach 642 million. The World Health Organization (WHO) estimates that diabetes consumes 12% of global medical expenses (approximately $673 billion). At present, the number of people with diabetes in China is approximately 110 million, and it has become the country with the largest number of patients with diabetes^3^. Additionally, the growth rate of direct diabetes medical expenses in China reached 19.90%, exceeding the growth rate of GDP and national total health expenditure during the same period. A study published by JAMA in 2017 showed that the average life expectancy of Chinese adult diabetic patients will be shortened by 9 years compared with those without diabetes^4^. Diabetes also poses a heavy economic burden on families and society while endangering human health. The initial symptoms of diabetes are relatively concealed, and it is difficult to confirm the diagnosis in the early stage of the disease. Patients are often diagnosed when the condition is serious or when there are one or more complications. According to the American Diabetes Association, early screening and detection in people with impaired blood glucose can delay the onset of diabetes through diet and exercise intervention^5^. In addition, early identification of high-risk groups of diabetes mellitus is also conducive to avoiding or controlling the development of complications.

In recent years, using data mining and machine learning methods to analyze collected medical data has attracted the interest of many researchers. At the same time, the accurate prediction of a disease outcome is one of the most challenging tasks for physicians. Some outstanding research has been successfully applied in breast cancer^6^, hepatocellular carcinoma^7^, lung cancer^8^, and other cancer types automatic recognition and survival prediction tasks^6–8^. Similar to cancer research, many machine learning methods have been proposed for the effective diagnosis, prognosis, management, and treatment of diabetes^9–11^ in the past few years. For the diagnosis and treatment of diseases, the methods used by researchers are divided into rule-based methods^12–15^, such as C4.5, CART, and random forest (RF), and nonrule-based methods^16–19^, such as support vector machine (SVM), Bayes, k-nearest neighbor (k-NN), and neural network (NN). When using a machine learning model to screen or diagnose individuals with diabetes mellitus, the model should not only have good discriminant performance but also ensure the transparency and interpretability of the discriminant process. Discriminant rules with causal logic can provide decision support for professionals, construct a model that can produce transparent discriminant rules, help people understand the internal mechanism of disease occurrence, and promote the development of disease research. Additionally, extracting and displaying the intrinsic discriminant rules of the model can help the expert question the discriminating mechanism. In addition, the transparent discrimination rule can also be checked twice by the expert to avoid errors and improve the reliability of the discrimination system^18^. Therefore, the interpretability of the model is of great value to ensure the usability of the diagnostic assistant system. The model used for diabetes diagnosis should have good interpretability and produce clear discriminant logic and intuitive results that can be easily understood by medical workers and patients.

To ensure the interpretability of the model, rule-based methods are usually used in existing research. The rule-based model compares its information gain based on the split value of a single feature. Although it is interpretable, it does not consider the correlation between features, which limits the discriminant performance of the model to a certain extent. The SVM is a classifier that maximizes the interval. The goal is to find a classification hyperplane that can separate samples of different categories. Due to the strong classification ability of SVM for medical data, SVM has been widely used in the diagnosis of various diseases, and its performance superiority has been confirmed^21–23^. However, SVM implicitly maps the input to the high-dimensional feature space in the process of calculating the hyperplane used for classification, which destroys the actual physical meaning of the input feature, resulting in the lack of interpretability, and it cannot clearly show the discriminant criteria and process. Therefore, SVM is generally used as a “black box”, which reduces its practical value, especially in the field of disease diagnosis. Considering the poor interpretability of the SVM model, the rule extraction method can be generally used to transform the model decision process into a rule set to improve the interpretability of the model.

This paper proposes a method for extracting the reduced rule from the fuzzy SVM and the biased random forest (BRF). First, we build the fuzzy SVM model with acceptable accuracy and extract the support vectors (SVs) from the SVM. Then, the fuzzy SVM is used to predict the SV labels. The SVs and predicted labels make up the artificial dataset. The artificial dataset is provided to theBRF to generate rules. Finally, the rule reduction module is introduced to remove redundant conditions and rules and improve the interpretability of the obtained rules.

The experimental results show that the proposed method generates more succinct and accurate rules than other methods, which is helpful for a broader assessment of diabetic patients. In addition, the results of the study indicate that the method can be used as a tool to detect diabetes and its associated risk factors. In summary, this work has the following major contributions:

- Developing a hybrid framework based on reduced rules extracted by BRF.
- It is proposed to utilize BRF to deal with the problem of data imbalance caused by diabetic patients far less than normal people.
- A reduction method based on the error rate and coverage rate is developed to remove the problems of similar, repetitive, and inefficient conditions and rules caused by the independent learning of each tree in the ensemble method.

The rest of this paper is organized as follows. The second section discusses the related work of SVM rule extraction. In third section, first, the framework of the algorithm is introduced, and then the algorithm is introduced in detail. The fourth section introduces the dataset and the experimental process. In fifth section, the experimental results are discussed. Finally, the last section is the conclusion.

## Related work

To achieve early detection and early intervention of diabetic patients, many methods have been proposed in recent years. Nilashi et al. used the EM method to cluster data, applied the PCA method to reduce the data dimensionality, filtered out the potential noise, and applied CART to find the decision rules from diabetes data^11^. Patil et al. proposed the HPM method, using C4.5 to classify the data denoised by the k-means clustering algorithm^24^. Due to the tree structure of CART, C4.5, and other decision tree models, the classification process is transparent, but they are weak classifiers. To improve the classification effect, the model with stronger learning ability is used. SVM has attracted attention for the diagnosis of diabetes due to its excellent classification ability. Shen et al. proposed an SVM parameter adjustment method using a fruit fly optimization algorithm and applied it to diabetes diagnosis^25^. It was verified that the method can obtain more suitable model parameters and greatly reduce the calculation time compared with other SVM parameter adjustment methods. Santhanam et al. used k-means to remove noise data, used a genetic algorithm to find the best feature set, and used SVM as a classifier to classify the diabetes data^26^. Uzer et al. proposed using an artificial bee colony algorithm for feature selection to eliminate the influence of unimportant features on SVM classification results^27^. Choubey et al. compared the effects of SVM using different kernel functions in the diagnosis of diabetes and used genetic algorithms to eliminate redundant features to reduce calculation costs and improve classification accuracy^28^.

SVM has a rigorous statistical learning theoretical basis, which can better solve the problems of overfitting, local minima, and dimension disasters. However, its classification process is not transparent, and it is used as a black box, which reduces its reliability. Rule extraction is an effective technique to solve this problem. At present, rule extraction for SVM can be divided into three categories: decomposition methods, pedagogical methods and eclectic methods^29^. The basic idea of the decomposition method is to decompose the SVM into several sets in units of SVs, search and extract rules for each SV, and finally combine these rules, such as SVM + prototype^30^ and HRE^31^. The pedagogical method does not consider the type and structure information of the SVM, ignores the knowledge provided by the SVs or decision boundary of the SVM, only pays attention to the mapping result of the SVM input–output, and uses the SVM as a "black box" to extract rules from the SVM prediction labels by the rule generation method. Other machine learning algorithms are used to extract rules, such as the GEX and G-REX algorithms, which generate rule sets using algorithms such as C4.5, CART, and Bayesian trees^32^. The advantage of this algorithm is that it is highly versatile. It is different from the decomposition method, which is usually applied to the linear SVM model. The pedagogical method is not limited by the type and structure of the SVM. However, the rule set is too large due to the use of all data generation rules. The eclectic method combines the advantages of the pedagogical method and the decomposition method, makes full use of the SV information in the SVM, and can also use a rule generation model to extract rules. To some extent, the SVM decision function information is considered, and the number of generated rules is also reduced. Han et al. proposed the SVM + RF algorithm, which uses random forests to generate rules from artificial datasets constructed from SVs^33^. The rules extracted by this method have good accuracy. However, the rules generated by the ensemble method are similar or even repeated, which harms the interpretability. Liu et al.^34^ and Khanam et al.^35^ used CART to extract rules from the SVM. Deshmukh et al.^36^ developed a hybrid fuzzy deep learning approach for the detection of diabetes. Firstly, the data was fuzzified. After that, a 5 × 5 fuzzy matrix was constructed. Lastly, the fuzzy matrix was fed into the convolution neural network (CNN).The results demonstrated that the fuzzified CNN approach outperformed the traditional NN approach. Azad et al.^37^ proposed a model PMSGD to classify diabetes. Synthetic minority over-sampling technique (SMOTE), genetic algorithm (GA), and DT were used in the proposed model. Wang et al.^38^ deleted the repeated rules and the repeated conditions in the rules to obtain a more concise rule set. Hayashi et al.^39^ proposed to combine rule extraction algorithm and sampling selection technique to achieve interpretable and accurate classification rules for PID data set. Similarly, Chakraborty et al.^40^ proposed the eclectic rule extraction from neural network recursively (ERENNR) algorithm, which generated rules from dataset with mixed attributes in the guise of attribute data ranges.

Overall, Han et al. noted that the eclectic method can reduce the degree of imbalance in the dataset^33^, but the effect is limited. Most of the existing rule extraction methods do not consider how to deal with the imbalance problem that is prevalent in medical datasets. In addition, the rules extracted by ensemble learning methods are redundant, which improves the risk of model overfitting. Using a decision tree to extract rules, because the model is generated by heuristic learning, there is a problem that cannot effectively minimize the global training error. To solve the above problems, a method for extracting reduced rules from SVM based on biased random forest is proposed.

## Proposed method

**Figure 1:**
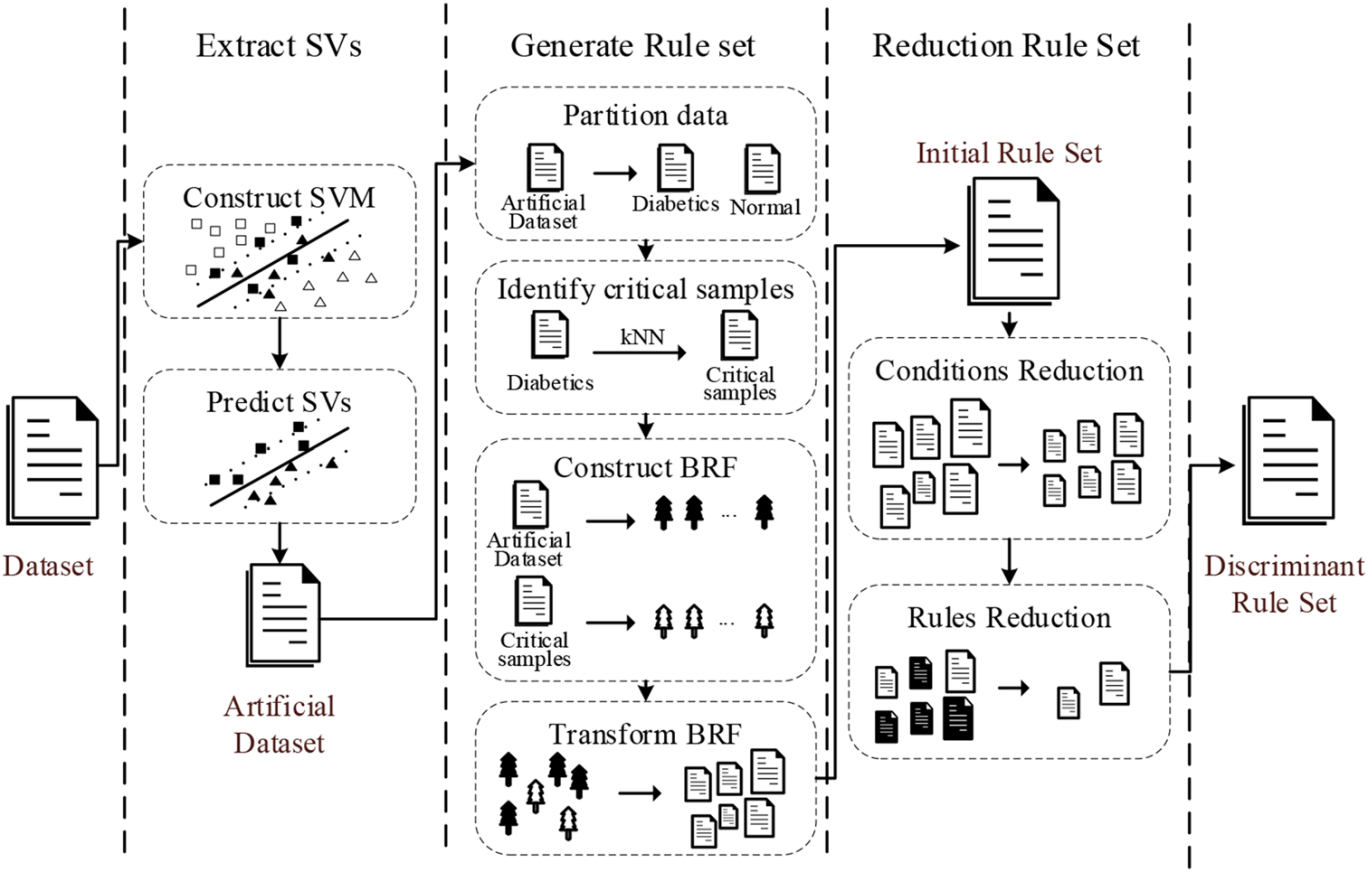
Schematic diagram of the proposed method.

In this section, the proposed rule extraction method is introduced. Figure 1 shows the algorithmic principle of the method for extracting reduced rules from SVM based on biased random forest. First, the SVM model is constructed by using the data preprocessed training set, and the hyperparameters are tuned to make the model have acceptable classification performance. Extracting the SVs, the richest information points containing partitioning patterns from SVM. The SVs are predicted by the trained SVM to obtain the labels. The SVs and their labels make up the artificial data to eliminate label noise. Then, the potential distribution of the artificial dataset is inferred through BRF, and each tree is traversed from the root node to the leaf node to generate “if–then” rules. Finally, the rule set generated by the BRF is reduced to obtain the discriminant rule set.

### Extract SVs

The purpose of the fuzzy SVM is to find the optimal hyperplane that can separate samples of different classes, while the hyperplane meets the constraints of maximizing samples and hyperplane spacing. In essence, fuzzy logic is used to classify the level of risks from data, SVM is used to design the fuzzy rules, and the dataset is used to train the SVM using Linear Parameter and test the fuzzy system. Finding the classification hyperplane can be transformed into a convex optimization problem:

1 $$\underset{{\varvec{w}},b,\xi }{min}\frac{1}{2}||{\varvec{w}}|{|}^{2}+C\sum_{i}{\xi }_{i}\quad\mathrm{ s}.\mathrm{t}.{y}_{i}(({\varvec{w}}{)}^{T}\varphi ({{\varvec{x}}}_{i})+b)\ge 1-{\xi }_{i},i=\mathrm{1,2},...,N$$

${\xi }_{i}i$ is a relaxation variable, which converts hard interval maximization into soft interval maximization. $C$ is the penalty factor to represent the penalty size of the misclassified samples. $\varphi (\cdot )$ indicates that the kernel technique is used to map the input space into the high-dimensional space, which can transform the linear indivisible problem into a linearly separable solution problem in high-dimensional space. Spatial mapping is usually implemented by the radial basis function (RBF):

2 $$K({\varvec{x}},{{\varvec{x}}}^{^{\prime}})=\mathrm{exp}(-\frac{||{\varvec{x}}-{{\varvec{x}}}^{^{\prime}}|{|}^{2}}{2{\sigma }^{2}})$$

$||{\varvec{x}}-{{\varvec{x}}}^{^{\prime}}|{|}^{2}$ represents the Euclidean distance between two vectors. $\sigma$ is a tunable parameter; the smaller $\sigma$ is, the more SVs there are, and the easier the model is overfitted.

To simplify the solution, the Lagrange multiplier ${\alpha }_{i}$ is introduced. By using the Lagrange dual property, the solution of Formula (1) is transformed into its dual problem:

3 $$\begin{gathered} \mathop {{\text{max}}}\limits_{\alpha } \mathop \sum \limits_{i = 1}^{N} \alpha_{i} - \frac{1}{2}\mathop \sum \limits_{i = 1,j = 1}^{N} \alpha_{i} y_{i} \alpha_{j} y_{j} K({\varvec{x}}_{i} ,{\varvec{x}}_{j} ) \hfill \\ {\text{s}}.{\text{t}}.C \ge \alpha_{i} \ge 0,i = 1,2, \ldots ,N \hfill \\ \mathop \sum \limits_{i = 1}^{N} \alpha_{i} y_{i} = 0 \hfill \\ \end{gathered}$$

The gradient descent method is used to solve ${\alpha }_{i}$. Then, the SVM classification decision function can be written as:

4 $$f({\varvec{x}})=\mathrm{sign}({\sum }_{i=1}^{sv}{\alpha }_{i}^{*}{y}_{i}K({{\varvec{x}}}_{i},{\varvec{x}})+b)$$

SVsAn SV is a sample of training data corresponding to a Lagrange multiplier greater than 0. Formula (4) shows that the discriminant result of the SVM discriminant model for new samples is entirely determined by SVs, and discriminant rule set extraction using SVs can retain the discriminant effect of the SVM model to a large extent. Through Formula (4), the researchers can prove that the rules in SVM are implied in SVs or decision boundaries. Therefore, rule extraction from SVM can be transformed into rule extraction from SVs. The complexity of computation depends on the number of SVs, not the dimension of the sample space, which avoids the “dimension disaster” in a sense and reduces the number of rules generated by rule extraction. It is worth noting that to strengthen the output accuracy, fuzzy SVM is used to optimize the traditional SVM classifier. Fuzzy SVM is able to emphasize the support vector node to avoid any redundant training since the crisp sets will be converted to fuzzy sets.

### Generation rule set

**Figure 2:**
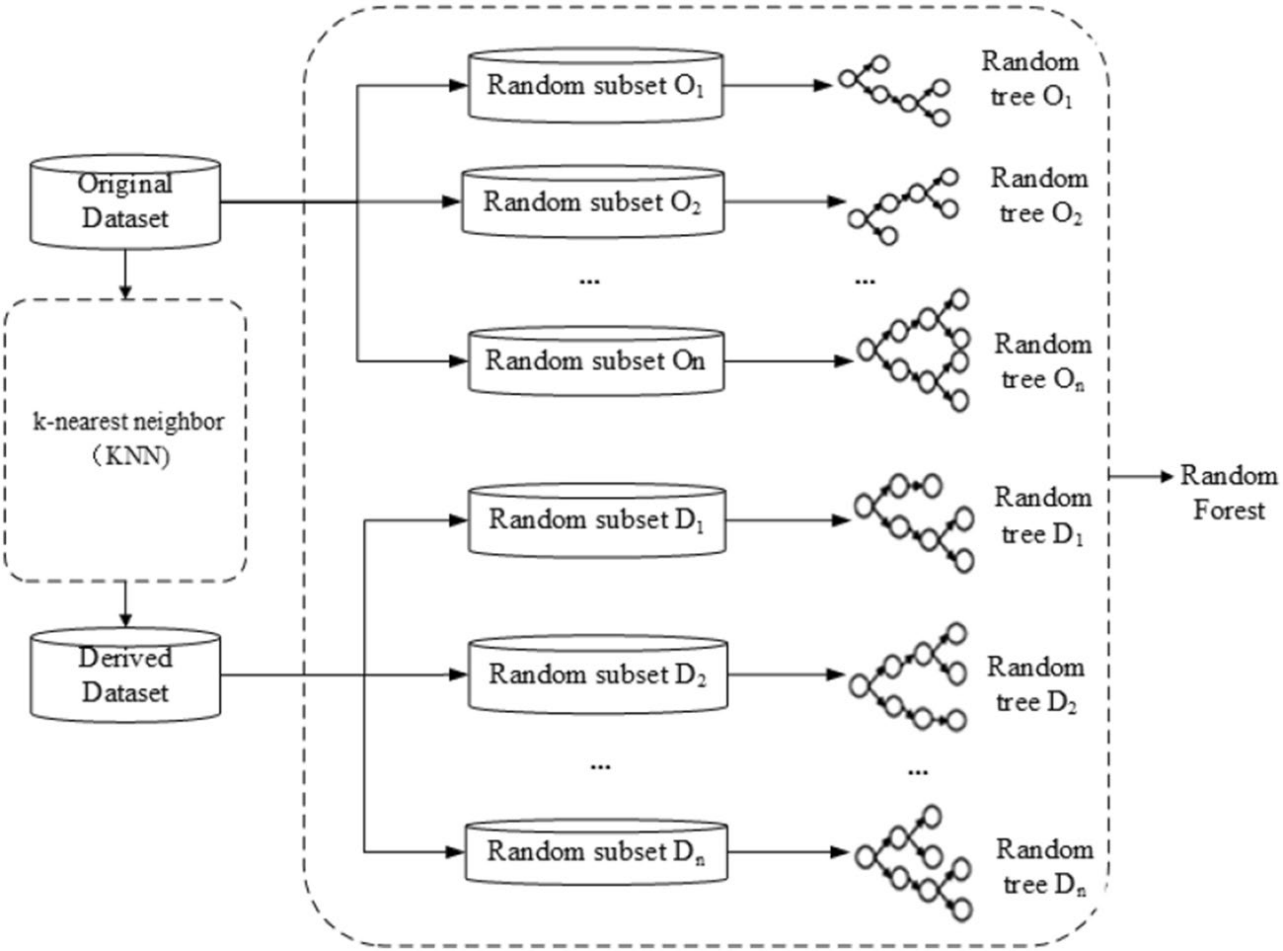
Schematic diagram of BRF.

Figure 2 shows the schematic diagram of BRF. It is an ensemble method to alleviate the data imbalance by increasing the number of classifiers representing the minority class^41^. Compared with RF, BRF defines the minority samples and their k-nearest neighbors as critical samples. For this part of the samples, more tree models are generated for classification. Move the sampling operation from the data level to the model level to obtain better results in imbalanced data classification. In the diagnosis of diabetes, the number of diabetic patients is far less than that of healthy people, which leads to an imbalance of the collected dataset. Although in the previous step, the imbalance problem of the artificial dataset constructed by SVs is slightly alleviated compared with that of the training dataset, the problem still exists and cannot be ignored. Taking advantage of BRF to generate rule sets is better than other ensemble learning methods due to its adaptability to imbalanced data.

Specifically, the dataset is first divided into a majority class set (normal) and a minority class set (diabetics). Then, the k-NN algorithm is used to find the k-nearest neighbors in the majority class set for each sample in the minority class set. If one sample in the majority class set appears repeatedly, only one is retained. The minority class set and the k-nearest neighbors in the majority class set form a new dataset. In addition to using the undivided dataset to build a random forest, the new dataset is also used to build a random forest. These forests are combined to obtain the final BRF. BRF can be seen as a method to learn from the original dataset and the undersampling subdataset generated from the original dataset. This kind of bias to the minority class compensates for its low presence in the dataset to overcome the data imbalance problem.

Rule generation is divided into two steps. First, the BRF model is induced based on an artificial dataset. Then, according to the BRF model, each tree is searched from the root node to the leaf node to extract the rules. The rules extracted from all trees are combined to form the initial rule set.

### Reduction rule set

The rules contained in the initial rule set have the problem of redundancy. The problem increases the risk of the rule set overfitting and affects the practicability of the rule set. Therefore, it is necessary to simplify the rule set. The reduction includes two steps: the first step is to remove the redundant conditions, and the second step is to reduce the redundant rules.

First, let the initial rule set be ${\mathfrak{R}}_{initial}=\{{R}_{i}\to {L}_{i},i=\mathrm{1,2},...,K\}$, where $K$ is the number of rules, ${R}_{i}$ is the $i$ th discrimination rule, and ${L}_{i}$ is the label corresponding to the $i$ th rule. Rules consist of multiple conditions, such as ${f}_{1}={v}_{1}\&{f}_{2}\le {v}_{2}\&{f}_{3}\ge {v}_{3}\cdots \&{f}_{j}\ge {v}_{j}\to 1$, where ${f}_{j}$ represents the $j$ th attribute in the rule, and ${v}_{j}$ represents the value of ${f}_{j}$. The pruning rule ${R}_{i}$, according to the removal of a certain condition, calculates the change in the error rate of rule ${R}_{i}$ to the sample to determine whether the condition should be removed, and the specific calculation formula is as follows:

5 $${D}_{j}=\frac{{err}_{-j}-{err}_{0}}{\mathrm{max}({err}_{0},s)}$$

In the formula, ${err}_{0}$ and ${err}_{-j}$ indicate the discrimination error rate of rule ${R}_{i}$ before and after, respectively, the $j$th condition is removed. It should be noted that the discriminant error rate of the rule is the proportion of the misjudged samples in the samples satisfying the rule. $s$ is a normal number to constrain the size of ${D}_{j}$. Set a threshold value (0.05 here). If ${D}_{j}$ is less than the threshold value, it denotes that the $j$th condition has little impact on the discrimination. It should be removed from ${R}_{i}$ and updated with ${err}_{0}$. Otherwise, the condition is kept, and the next condition is evaluated. After all the rules in the initial rule set are processed, the conditions reduced rule set $\mathfrak{R}=\{{R}_{i}^{^{\prime}}\to {L}_{i},i=\mathrm{1,2},...,K\}$ is obtained, where ${R}_{i}^{^{\prime}}$ is the reduced rule ${R}_{i}$.

The next step is to reduce the redundant rules. First, an empty set ${\mathfrak{R}}_{final}=\{\}$ is constructed to store the filtered rule set. Then, the rule set $\mathfrak{R}$ is roughly screened by rule coverage, which is expressed as:

6 $$freq=\frac{{N}_{{R}_{i}^{^{\prime}}}}{N}$$

where ${N}_{{R}_{i}^{^{\prime}}}$ represents the number of training samples that meet rule ${R}_{i}^{^{\prime}}$, and $N$ represents the total number of training samples. Set the threshold $g$, and remove the rules whose coverage is less than $g$ from $\mathfrak{R}$. At the same time, a default rule ${R}_{def}=\{\}\to {L}^{*}$ is built, where ${L}^{*}$ represents the label with the largest number of samples in the training set. Remove the rules with low coverage in rule set $\mathfrak{R}$, and add ${R}_{def}$ to form rule set ${\mathfrak{R}}^{^{\prime}}$. Then, the training dataset and rule set ${\mathfrak{R}}^{^{\prime}}$ are used to filter the rules iteratively, in which rule ${R}_{best}$ with the minimum discrimination error rate is selected into ${\mathfrak{R}}_{final}$ for each iteration, the samples satisfying rule ${R}_{best}$ are removed from the training dataset, ${R}_{best}$ is removed from ${\mathfrak{R}}^{^{\prime}}$, and the output label ${L}^{*}$ of the default rule ${R}_{def}$ is updated according to the updated training dataset. Finally, when the rule ${R}_{best}$ selected is ${R}_{def}$ or the training dataset is empty, the iterative process of rule filtering is stopped. If ${R}_{best}$ is the default rule, add the default rule to ${\mathfrak{R}}_{final}$. If the training dataset is empty, update the output label of the default rule to the initial value, and add the rule to ${\mathfrak{R}}_{final}$. ${\mathfrak{R}}_{final}$ is the final set of reduced discriminant rules. The pseudocode for reducing the redundant rules is shown in Table 1.

**Table 1:**
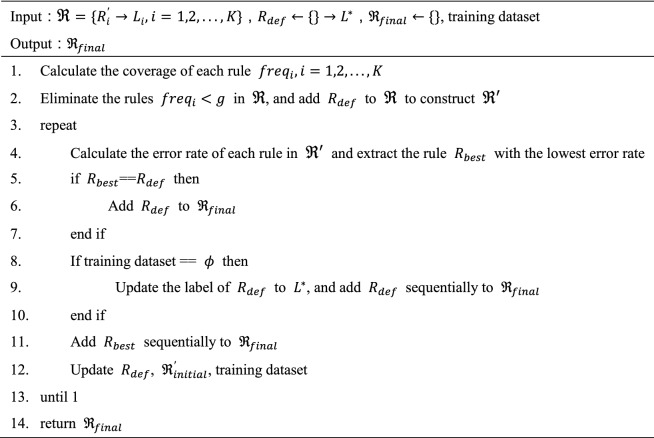
Pseudo-code of reduce the redundant rules.

## Experiments

In this study, a new interpretability approach for rule extraction from the fuzzy SVM is proposed. This technology integrates the information provided by the SVs of the SVM model into the BRF method to extract rules from the black box SVM model and reduces the conditions and rules to improve interpretability. First, to verify the rule extraction motivation from the SVM, the SVM is compared with the RF, C4.5, ID3, CART, and RIPPER methods. Then, SVM + BRF (not reduced) and fuzzySVM + BRF (not reduced) are compared with SVM + RF^33,35^. Finally, the proposed method is compared with Re-RX + J48graft(2016)^36^, Fuzzy + CNN(2019)^33^, ERENNR(2019)^37^, SVM + XGBoost(2019)^17^, RF + XGBoost(2021)^16^, and PMSGD(2019)^34^ methods. Finally, all methods are tested on the test set.

### Dataset

The experimental data are the Diabetes Medical Examination Data collected by Beijing Hospital (DMED-BH). The DMED-BH consists of 17 features, which can be divided into noninvasive and invasive features, including routine physical examination indicators, blood test indicators and questionnaire survey indicators. According to WHO’s definition of diabetes, the fasting blood glucose $\ge$ 7.0 mmol/L and/or the postprandial blood glucose $\ge$ 11.1 mmol/L, 6503 people are marked as diabetes positive and 36,853 people are marked as diabetes negative.

### Experiment environment

Table 2 shows the experiment environment. Each model is implemented using Tensorflow v2.2.0 and trained by a personal computer with Intel i7-8750 CPU, 8 GB RAM, Windows 10 operating system.

**Table 2:** Experiment environment.

Environment	Description
Computer	Intel(R) Core(TM) i7-8750H CPU
Application platform	Windows10
Software	R 4.0.3 Tensorflow v2.2.0

### Evaluation metrics

The primary goal of rule extraction is to improve the interpretability of the model while ensuring the discriminant ability of the model. In particular, in the diagnosis of diabetes, we cannot blindly pursue interpretability without considering the accuracy of the discriminant results. For all methods, accuracy, precision, recall, F1- measure and Mathews correlation coefficient (MCC) are used as evaluation metrics in the experiment, and the number of rules is also used to measure the interpretability of rules. Formulas for these metrics are shown in Eqs. (7)–(11).

7 $$Accuracy= \frac{TP+TN}{TP+TN+FP+FN}$$

8 $$Precision= \frac{TP}{TP+FP}$$

9 $$Recall= \frac{TP}{TP+FN}$$

10 $$F1= \frac{2\times Precision\times Recall}{(Precision+Recall)}$$

11 $$MCC= \frac{TP\times TN-FP\times FN}{\sqrt{(TP+FN)\times (TN+FP)\times (TP+FP)\times (TN+FN)}}$$

where $TP$ indicates the true positive frequency, $FP$ indicates the false positive frequency, $TN$ indicates the true negative frequency, and $FN$ indicates the false negative frequency. $F1$ is the weighted harmonic average of $precision$ and $recall$ and gives them the same weight. $MCC$ is considered to be a relatively balanced metric, which can be applied even when the data are imbalanced.

### Feature selection

Many machine learning methods may lead to worse performance because of a large number of redundant features. Feature selection has important practical significance^42^. It not only reduces overfitting, reduces the number of features, and improves the generalization ability of the model but also accelerates the training speed of the model. Generally, feature selection can improve the model performance. Therefore, the filtering method and embedding method are used for feature selection. Among them, the filtering method uses the chi square test and information gain, and the embedding method is realized by RF.

The chi square test is one of the commonly used methods for feature selection to determine whether the two variables are independent by observing the deviation between the actual value and the theoretical value^43^. In addition to the chi square test, information gain is also a very effective feature selection method. Unlike the chi square test, which uses correlation between features and labels to quantify the importance of features, information gain is based on the amount of feature information^44^. Random forest is a typical ensemble learning method that is often used for feature selection^45^. The idea is to compare the contribution of each feature in random forest; the greater the contribution is, the more important the feature. Generally, the Gini index is used to measure the contribution of features^46^.

Considering the effect and efficiency of diabetes diagnosis, the features evaluated by the chi square test, information gain and RF are ranked, and the average rank is calculated. The top 9 features with the highest average rank and statistical significance (p value < 0.05) were selected to build the models. They are AGE, WEIGHT, HEIGHT, CHOL (cholesterol), TG (triglyceride), HDL (high-density lipoprotein), LDL (low-density lipoprotein), SBP (systolic blood pressure) and DBP (diastolic blood pressure). The result of feature selection is shown in Table 3.

**Table 3:** Feature selection results.

Features	Chi square (p-value)	IG	RF
AGE	140.5 (0.0000)	0.071	12.399
WEIGHT	559.45 (0.0000)	0.259	10.529
HEIGHT	392.51 (0.0000)	0.192	10.224
CHOL	406.19 (0.0021)	0.196	9.844
TG	415.31 (0.0000)	0.201	11.041
HDL	221.72 (0.0000)	0.118	13.174
LDL	391.37 (0.0001)	0.190	10.617
SBP	175.24 (0.0000)	0.092	10.855
DBP	108.89 (0.0000)	0.056	9.998

### Rule extraction performance

To obtain reliable and stable models, fivefold cross validation (fivefold CV) is used to determine the model parameters and test models. The dataset is randomly and evenly divided into 5 parts, one of which is used as the test set, one of which is used as the validation set, and the remaining three parts are used as the training set. The training set is used to train the SVM, the validation set is used to evaluate the performance of the model under different hyperparameters, and the test set is used to evaluate the performance of the SVM using the hyperparameters that perform best on the validation set. First, through grid search, the optimal hyperparameters (gamma and cost) of the SVM are 1.5 and 4. It is worth noting that the SVM uses the radial basis function (RBF) as the kernel function and normalizes the data to [0,1] during training. Then, the SVM is trained on the new training set consisting of the training set and the validation set, and the test results are obtained on the test set. This process is also carried out in fivefold CV.

In addition, to prove the motivation for extracting rules from SVM, RF, C4.5, ID3, CART and RIPPER are used as the comparison methods. As with SVM, these methods are adjusted by fivefold CV to obtain test results. The performance of these models is evaluated by accuracy, precision, recall, F1-measure and MCC. The results are shown in Table 4.

**Table 4:** Average results of fivefold CV for positive class.

Methods	Accuracy (%)	Precision (%)	Recall (%)	F1	MCC
SVM	**98.84**	**98.67**	**61.77**	**0.7496**	**0.7472**
RF	92.70	92.36	60.54	0.7351	0.7210
C4.5	50.60	41.52	40.77	0.4061	0.3088
ID3	46.81	45.76	37.69	0.4049	0.3285
CART	44.86	44.09	28.46	0.3361	0.2624
RIPPER	49.99	49.21	22.31	0.2705	0.2391

Significant values are in bold.

In the process of fivefold CV, SVs are extracted from the trained SVM model. The average number of SVs is 653.5 (standard deviation is 4.394), and the average ratio of positive and negative diabetes in SVs is 1:5.45 (standard deviation is 0.406), which is slightly lower than the ratio of 1:5.7 in the original dataset, but the imbalance problem still exists. This is the motivation for using the BRF method, which can effectively deal with the imbalance problem to extract rules. The SVs and prediction results via the SVM are combined into an artificial dataset. The new dataset is used to extract rules from the SVM, training rule-based learners to obtain rules that can express the connotation of the SVM. RF, which is an ensemble method similar to BRF, is used as a comparison method. The results are shown in Table 5.

**Table 5:** Average results of fivefold CV for ensemble methods.

Methods	Accuracy (%)	Precision (%)	Recall (%)	F1	MCC
SVM + RF	91.96	89.87	83.77	0.8671	0.8258
SVM + BRF(ours)	**96.84**	**94.78**	**90.08**	**0.9237**	**0.8428**
FuzzySVM + BRF(ours)	**98.73**	**96.83**	**93.01**	**0.9488**	**0.8524**

Significant values are in bold.

The rules obtained from BRF are reduced by the method in Sect. 3.3. The reduced rule sets (FuzzySVM + BRF + reduced, SVM + BRF + reduced) are compared with the rule sets reduced by the Re-RX + J48graft(2016)^36^, Fuzzy + CNN(2019)^33^, ERENNR(2019)^37^, SVM + XGBoost(2019)^17^, RF + XGBoost(2021)^16^, and PMSGD(2019)^34^ methods. We tested these comparison methods on DMED-BH dataset. In addition to using accuracy, precision, recall, F1-measure and MCC to evaluate the rule set performance in the diagnosis of diabetes, the number of rules is also used to represent the interpretability of rules. The results are shown in Table 6.

**Table 6:** Average results of fivefold CV for extracted rule sets on DMED-BH dataset.

Methods	Accuracy (%)	Precision (%)	Recall (%)	F1	MCC	Rules
Re-RX + J48graft(2016)^36^	83.96	83.25	85.38	0.8430	**0.8726**	**8.2 ± 1.0**
Fuzzy + CNN(2019)^33^	**94.74**	**94.94**	**93.02**	**0.9397**	0.8626	29.3 ± 1.1
ERENNR(2019)^37^	83.71	81.25	83.96	0.8258	0.8617	72.4 ± 6.0
SVM + XGBoost(2019)^17^	90.89	89.53	82.26	0.8574	0.8534	13.4 ± 7.6
RF + XGBoost(2021)^16^	90.93	89.22	85.47	0.8730	0.8722	18.5 ± 2.9
PMSGD(2021)^34^	83.86	82.16	85.47	0.8378	**0.8759**	21.5 ± 3.7
SVM + BRF + reduced (ours)	**95.75**	**93.50**	**91.49**	**0.9248**	0.8653	**12.4 ± 2.5**
FuzzySVM + BRF + reduced (ours)	**96.92**	**94.81**	**93.11**	**0.9395**	**0.8802**	**9.2 ± 1.6**

Significant values are in bold.

### Generality analysis

To verify the generality of the proposed method, two open datasets related to diabetes were selected and tested. The selected datasets are described as follows:

Pima Indian Diabetes (PID)^47^. A PID dataset was used to test the effectiveness of various diagnostic methods for diabetes. There are 768 samples in the dataset (268 cases 1 and 500 cases 0), and the ratio of positive samples to negative samples is 1:1.87. Each sample is represented by 8 features: pregnancy, glucose, blood pressure, skin thickness, insulin, BMI, diabetes pedigree function, and age.

China Health and Nutrition Survey (CHNS)^45,46^. The CHNS dataset was collected by the Chinese Center for Disease Control and Prevention and the Carolina Population Center at the University of North Carolina at Chapel Hill. The dataset we selected was collected in 2009, covering nine provinces in China. After data preprocessing, there were 7,913 samples. The samples with fasting blood glucose ≥ 7.0 mmol/L were labeled positive samples, and the ratio of positive samples to negative samples was 1:11.2. After feature selection, a total of 9 features were used in the experiment: WEIGHT, AGE, WAIST, DBP, CHOL, TG, HBA1C, UA, and HDL.

Five-fold cross validation was carried out according to the process in “Feature selection”, and some experimental results were extracted from their original paper. The summarized experimental results are shown in Tables 7 and 8.

**Table 7:** Average results of fivefold CV for extracted rule sets on PID dataset.

Methods	Accuracy (%)	Precision (%)	Recall (%)	F1	MCC	Rules
Re-RX + J48graft(2016)^36^	84.93	83.83	78.64	0.8115	**0.8796**	**8.2 ± 2.1**
Fuzzy + CNN(2019)^33^	**95.74**	**95.00**	**92.01**	**0.9348**	0.8355	28.9 ± 9.5
ERENNR(2019)^37^	84.71	83.12	81.56	0.8233	0.8518	79.1 ± 6.9
SVM + XGBoost(2019)^17^	76.77	75.32	73.62	0.7446	0.7747	23.2 ± 2.7
RF + XGBoost(2021)^16^	89.60	88.32	86.55	0.8742	0.8578	19.4 ± 0.8
PMSGD(2021)^34^	83.64	82.13	80.09	0.8109	0.84026	24.7 ± 3.1
SVM + BRF + reduced (ours)	**95.92**	**94.78**	**92.95**	**0.9385**	**0.8746**	**18.2 ± 1.5**
FuzzySVM + BRF + reduced (ours)	**96.84**	**95.23**	**93.64**	**0.9442**	**0.8752**	**8.9 ± 1.3**

Significant values are in bold.

**Table 8:** Average results of fivefold CV for extracted rule sets on CHNS dataset.

Methods	Accuracy (%)	Precision (%)	Recall (%)	F1	MCC	Rules
Re-RX + J48graft(2016)^36^	83.59	80.87	79.56	0.8020	0.7794	**7.6 ± 2.0**
Fuzzy + CNN(2019)^33^	**93.46**	**92.83**	**92.22**	**0.9252**	**0.8857**	23.5 ± 8.0
ERENNR(2019)^37^	84.76	83.21	82.30	0.8275	0.8227	76.4 ± 3.2
SVM + XGBoost(2019)^17^	81.91	79.37	71.54	0.7525	0.7790	24.5 ± 2.8
RF + XGBoost(2021)^16^	88.64	87.36	87.55	0.8745	0.8401	19.4 ± 0.8
PMSGD(2021)^34^	90.85	89.15	83.94	0.8646	**0.8775**	17.8 ± 2.1
SVM + BRF + reduced (ours)	**93.62**	**92.78**	**91.44**	**0.9210**	0.8122	**13.3 ± 0.7**
FuzzySVM + BRF + reduced (ours)	**94.92**	**92.87**	**92.98**	**0.9292**	**0.8956**	**7.2 ± 1.6**

Significant values are in bold.

## Discussion

The main purpose of this study was to achieve a diabetes diagnosis. The models and rule sets are evaluated by accuracy, precision, recall, F1-measure and MCC. Among them, in the disease diagnosis field, false negatives need to be minimized, and the dataset has the characteristics of class imbalance, so recall and MCC should be given priority^48,49^.

In Table 4, compared with rule-based classifiers such as RF, C4.5, ID3, CART, and RIPPER, SVM has the highest accuracy, precision, recall rate, F1-measure, and MCC, which proves that SVM has better performance than the rule-based models. The results also demonstrate the rationality of our motivation to choose SVM as the basic classifier for diabetes detection. In Table 5, the rule sets extracted by BRF are superior to the rule sets extracted by RF in all indicators. After fuzzy logic is combined, our method achieves a better separation effect. Since fuzzy SVM can highlight support vector nodes to minimize duplicate training and meet the goal of improving output accuracy. In Table 6, compared with the six rule extraction models, except the fuzzy + CNN method, our method has obvious advantages in accuracy, precision, recall rate, F1-measure, and the number of MCC and reduction rules. Furthermore, while the fuzzy + CNN method has high accuracy, precision, and recall rate, the classifier tends to select the majority classes due to the naturally imbalanced character of the diabetes dataset. As a result, these indicators cannot accurately reflect the classifier's performance. Because MCC has little to do with the distribution of positive and negative samples, we focus more on MCC value comparison. In this way, fuzzy SVM + BRF outperforms fuzzy + CNN. It is worth mentioning that although the PMSGD method does not have high accuracy and rule reduction effect, it also has good classification performance on imbalanced data sets. The rule reduction number of the Re-Rx + J48graft method is also ideal, but the classification effect is not as good as our method in the diabetes prediction task. Tables 7 and 8 provide similar experimental results to Table 6, indicating that the proposed method also performs well on different data sets, proving the method's generality.

In summary, the proposed method can adapt to imbalanced data and extract rules that tend to diagnose patients with diabetes and further enhance interpretability by reducing rules. It is an effective method to extract rules from SVM for diabetes diagnosis.

Needless to say, the diagnosis of diabetes remains a complex problem; therefore, the fuzzySVM + BRF method should be tested on more recent and complete diabetes datasets in future studies to ensure that the most highly accurate rules can be extracted for diagnosis.

## Conclusion

Diabetes mellitus is a common chronic disease that seriously endangers human health. In recent years, machine learning methods have been widely used in diabetes diagnosis. Fuzzy SVM can emphasize support vector nodes, avoid redundant training, and simplify classification without sacrificing classification accuracy. Although fuzzy SVM has achieved great discrimination effects, the lack of interpretability due to mapping features to high-dimensional spaces during the classification process limits its application in the field of disease diagnosis. Therefore, it is necessary to extract rules for SVM. Considering the poor adaptability of the existing rule extraction methods to imbalanced data, the extracted rules tend to identify healthy people, and the BRF with a reduction module was proposed for rule extraction to solve the problem. First, the support vectors are extracted from the SVM model with acceptable classification ability, and the SVM is used to predict the support vectors. The support vectors and prediction results constitute an artificial dataset. Then, the critical samples are defined by the k-NN algorithm. Based on the critical samples, more trees are generated to be a part of the BRF. BRF is used to infer the potential distribution of the artificial dataset and obtain the initial rule set. Finally, the rule set is reduced to obtain the final rule set. The extracted rule set provides a basis for early intervention measures for diabetic patients and control of diabetes.

The experimental results show that the proposed model performs well in the four metrics of accuracy, recall, F1-measure, and MCC when the sizes of the rule sets are almost the same. This shows that the model is promising in diabetes diagnosis. A possible extension of this work is to consider how to generate the rule set to improve the accuracy, while maintaining recall.

## References

[1] Zhao M, Wang X, Zhu X (2014). Understanding diabetes from the diagnosis of diabetes mellitus [J]. J. Diagn. Concepts Pract..

[2] Cho, N., Whiting, D., & Forouhi, N. IDF Diabetes Atlas [R]. Brussels, Belgium: International Diabetes Federation (2016).

[3] Chinese Diabetes Society (2018). Guidelines for the prevention and control of type 2 diabetes in China [J]. Chin. J. Pract. Internal Med..

[4] Bragg F, Holmes MV, Iona A (2017). Association between diabetes and cause-specific mortality in rural and urban areas of Chine [J]. J. Am. Med. Assoc..

[5] Patil R, Tamane S, Rawandale SA (2022). A modified mayfly-SVM approach for early detection of type 2 diabetes mellitus[J]. Int. J. Electr. Comput. Eng..

[6] Montazeri M, Montazeri M, Montazeri M (2016). Machine learning models in breast cancer survival prediction[J]. Technol. Health Care.

[7] Książek W, Gandor M, Pławiak P (2021). Comparison of various approaches to combine logistic regression with genetic algorithms in survival prediction of hepatocellular carcinoma[J]. Comput. Biol. Med..

[8] Doppalapudi S, Qiu RG, Badr Y (2021). Lung cancer survival period prediction and understanding: Deep learning approaches[J]. Int. J. Med. Informatics.

[9] Faura G, Boix-Lemonche G, Holmeide AK (2022). Colorimetric and electrochemical screening for early detection of diabetes mellitus and diabetic retinopathy—application of sensor arrays and machine learning[J]. Sensors.

[10] Choubey DK, Tripathi S, Kumar P (2021). Classification of diabetes by kernel based SVM with PSO[J]. Recent Adv. Comput. Sci. Commun..

[11] Dremin V, Marcinkevics Z, Zherebtsov E (2021). Skin complications of diabetes mellitus revealed by polarized hyperspectral imaging and machine learning[J]. IEEE Trans. Med. Imaging.

[12] Latchoumi TP, Dayanika J, Archana G (2021). A comparative study of machine learning algorithms using quick-witted diabetic prevention [J]. Ann. Roman. Soc. Cell Biol..

[13] Tiddi I, Schlobach S (2022). Knowledge graphs as tools for explainable machine learning: A survey[J]. Artif. Intell..

[14] Du Y, Rafferty AR, McAuliffe FM (2022). An explainable machine learning-based clinical decision support system for prediction of gestational diabetes mellitus [J]. Sci. Rep..

[15] Tama BA, Rhee KH (2019). Tree-based classifier ensembles for early detection method of diabetes: An exploratory study [J]. Artif. Intell. Rev..

[16] Kumari S, Kumar D, Mittal M (2021). An ensemble approach for classification and prediction of diabetes mellitus using soft voting classifier[J]. Int. J. Cognit. Comput. Eng..

[17] Farran B, AlWotayan R, Alkandari H (2019). Use of non-invasive parameters and machine-learning algorithms for predicting future risk of type 2 diabetes: A retrospective cohort study of health data from Kuwait [J]. Front. Endocrinol..

[18] Hasan MK, Alam MA, Das D (2020). Diabetes prediction using ensembling of different machine learning classifiers[J]. IEEE Access.

[19] Singh A, Dhillon A, Kumar N (2021). eDiaPredict: An Ensemble-based framework for diabetes prediction[J]. ACM Trans. Multimed. Comput. Commun. Appl..

[20] Singh N, Singh P, Bhagat D (2019). A rule extraction approach from support vector machines for diagnosing hypertension among diabetics [J]. Expert Syst. Appl..

[21] Thaiyalnayaki K (2021). Classification of diabetes using deep learning and svm techniques[J]. Int. J. Curr. Res. Rev..

[22] Jaiswal V, Negi A, Pal T (2021). A review on current advances in machine learning based diabetes prediction[J]. Prim. Care Diabetes.

[23] Almansour NA, Syed HF, Khayat NR (2019). Neural network and support vector machine for the prediction of chronic kidney disease: A comparative study [J]. Comput. Biol. Med..

[24] Patil BM, Joshi RC, Toshniwal D (2010). Hybrid prediction model for Type-2 diabetic patients [J]. Expert Syst. Appl..

[25] Shen L, Chen H, Yu Z (2016). Evolving support vector machines using fruit fly optimization for medical data classification [J]. Knowl.-Based Syst..

[26] Santhanam T, Padmavathi MS (2015). Application of K-Means and Genetic Algorithms for Dimension Reduction by Integrating SVM for Diabetes Diagnosis [C]. In proceedings of Graph algorithms, High performance implementations and its applications, India.

[27] Uzer, M. S., Yilmaz, N., & Inan, O. Feature selection method based on artificial bee colony algorithm and support vector machines for medical datasets classification [J]. *Sci. World J.* (2013). 10.1155/2013/419187 PMC3745978 23983632

[28] Choubey, D. K., & Paul, S. GA_SVM: A classification system for diagnosis of diabetes [M]. Handbook of research on soft computing and nature-inspired algorithms, 2017, 359–397.

[29] Barakar N, Bradley AP (2010). Rule extraction from support vector machines: A review [J]. Neurocomputing.

[30] Núñez, H., Angulo, C., & Català, A. Rule extraction from support vector machines [C]. In *proceedings of European Symposium on Artificial Neural Networks*, Bruges, Belgium, 2002, 107–112.D

[31] Zhang Y, Su H, Jia T (2005). Rule extraction from trained support vector machines [J]. Adv. Tech. Knowl. Discov. Data Min.

[32] Martens D, Baesens B, Van Gestel T (2007). Comprehensible credit scoring models using rule extraction from support vector machines [J]. Eur. J. Oper. Res..

[33] Han L, Luo S, Yu J (2015). Rule extraction from support vector machines using ensemble learning approach: An application for diagnosis of diabetes [J]. IEEE J. Biomed. Health Inform..

[34] Liu C, Soong R, Lee W (2018). A predictive model for acute allograft rejection of liver transplantation [J]. Expert Syst. Appl..

[35] Khanam JJ, Foo SY (2021). A comparison of machine learning algorithms for diabetes prediction[J]. ICT Express.

[36] Deshmukh, T., Fadewar, H. S., & Shukla, A. The detection of Prameha (diabetes) in Ayurvedic way with the help of fuzzy deep learning. In *International Conference on Intelligent Computing and Communication Technologies*, pp. 152–158 (Springer, Singapore, 2019).

[37] Azad C, Bhushan B, Sharma R (2021). Prediction model using SMOTE, genetic algorithm and decision tree (PMSGD) for classification of diabetes mellitus[J]. Multimed. Syst..

[38] Wang Y, Wang D, Geng N (2019). Stacking-based ensemble learning of decision trees for interpretable prostate cancer detection [J]. Appl. Soft Comput. J..

[39] Hayashi Y, Yukita S (2016). Rule extraction using Recursive-Rule extraction algorithm with J48graft combined with sampling selection techniques for the diagnosis of type 2 diabetes mellitus in the Pima Indian dataset[J]. Inf. Med. Unlocked.

[40] Chakraborty M, Biswas SK, Purkayastha B (2019). Rule extraction from neural network using input data ranges recursively[J]. N. Gener. Comput..

[41] Bader-El-Den M, Teitei E, Perry T (2019). Biased random forest for dealing with the class imbalance problem [J]. IEEE Trans. Neural Netw. Learn. Syst..

[42] Saeys Y, Inza I, Larranaga P (2007). A review of feature selection techniques in bioinformatics [J]. Bioinformatics.

[43] Jin, X., Xu, A., & Bie, R. Machine learning techniques and Chi-square feature selection for cancer classification using SAGE gene expression profiles [C]. In *proceedings of the 1st workshop on Data Mining for Biomedical Applications, Singapore*, 106–115.

[44] Brown, G. A new perspective for information theoretic feature selection [C]. In *Proceedings of the twelfth international conference on artificial intelligence and statistics (AISTATS)*, 49–56.

[45] Qi Y (2012). Random Forest for Bioinformatics [J]. Ensemble Mach. Learn..

[46] Menze BH, Kelm BM, Masuch R (2009). A comparison of random forest and its Gini importance with standard chemometric methods for the feature selection and classification of spectral data [J]. BMC Bioinf..

[47] Cheruku R, Edla D, Kuppili V (2019). An optimized and efficient radial basis neural network using cluster validity index for diabetes classification [J]. Int. Arab J. Inf. Technol..

[48] Zhang XT, Jiang YL, Hu WJ (2020). A parallel ensemble fuzzy classifier for diabetes diagnosis [J]. J. Med. Imaging Health Inf..

[49] Hu YX, Luo SL, Han LF (2020). Deep supervised learning with mixture of neural networks [J]. Artif. Intell. Med..

